# Irisin inhibits microglial senescence via TFAM-mediated mitochondrial metabolism in a mouse model of tauopathy

**DOI:** 10.1186/s12979-024-00437-0

**Published:** 2024-05-14

**Authors:** Cailin Wang, Xiufeng Wang, Shangqi Sun, Yanmin Chang, Piaopiao Lian, Hongxiu Guo, Siyi Zheng, Rong Ma, Gang Li

**Affiliations:** 1 Department of Neurology, Tongji Medical College, Union Hospital, Huazhong University of Science and Technology, Wuhan, 430022 China; 2 Department of Pharmacology, School of Basic Medicine, Tongji Medical College, Huazhong University of Science and Technology, Wuhan, 430030 China

**Keywords:** Tau, Irisin, Microglial senescence, TFAM, Oxidative phosphorylation

## Abstract

**Background:**

The accumulation of senescent microglia has been highlighted as a critical contributor to the progression of tauopathies. Irisin, a muscle-derived hormone produced by the proteolytic cleavage of Fibronectin-domain III containing 5 (FNDC5), mediates the pleiotropic effects of exercise on the physical body. Herein, we investigate the potential role of irisin in microglial senescence in tauopathies.

**Methods:**

To model tauopathies both in vivo and in vitro, we utilized P301S tau transgenic mice and tau K18 fibril-treated microglia BV2 cells, respectively. We first examined the expression of the irisin expression and senescence phenotypes of microglia in tauopathies. Subsequently, we investigated the impact of irisin on microglial senescence and its underlying molecular mechanisms.

**Result:**

We observed a reduction in irisin levels and an onset of premature microglial senescence both in vivo and in vitro. Irisin administration was found to counteract microglial senescence and ameliorate cognitive decline in P301S mice. Mechanistically, irisin effectively inhibited microglial senescence by stimulating the expression of mitochondrial transcription factor A (TFAM), a master regulator of mitochondrial respiratory chain biogenesis, thereby enhancing mitochondrial oxidative phosphorylation (OXPHOS). Silencing TFAM eliminated the inhibitory effect of irisin on microglial senescence as well as the restorative effect of irisin on mitochondrial OXPHOS. Furthermore, the SIRT1/PGC1α signaling pathway appeared to be implicated in irisin-mediated upregulation of TFAM.

**Conclusion:**

Taken together, our study revealed that irisin mitigated microglial senescence via TFAM-driven mitochondrial biogenesis, suggesting a promising new avenue for therapeutic strategies targeting tauopathies.

**Supplementary Information:**

The online version contains supplementary material available at 10.1186/s12979-024-00437-0.

## Introduction

Tauopathies are a spectrum of age-related neurodegenerative diseases featuring the abnormal aggregation of tau in the brain. Alzheimer’s disease (AD) is one of the most common tauopathies, affecting millions of people globally [1, 2]. As a microtubule-associated protein, tau physiologically stabilizes microtubules and supports neuronal function. In tauopathies, however, hyperphosphorylated tau protein leads to its misfolding and subsequent formation of neurofibrillary tangles, resulting in chronic neuroinflammation, neuronal loss, and cognitive decline. Despite significant research efforts, the precise mechanisms underlying tau-induced neurotoxicity remain to be fully clarified.

In recent years, the role of premature cellular senescence in the initiation and progression of tauopathies has been highlighted across multiple studies [3–6]. Cellular senescence is a state of permanent cell cycle arrest in response to diverse damaged stimuli. Notably, senescent cells secrete various pro-inflammatory cytokines, chemokines, and growth factors into surrounding areas, referred to as the senescence-associated secretory phenotype (SASP). While senescent cells play beneficial roles in embryogenesis, wound healing, and tumor suppression, the accumulation of senescent cells during aging fosters a pro-inflammatory milieu and promotes paracrine senescence, ultimately leading to detrimental consequences for the organism [7, 8].

Microglia, the most abundant resident brain macrophages, comprise 10–15% of all glial cells and serve as primary defense against cerebral insults [9]. Microglia can rapidly expand their processes in the healthy brain to engulf and degrade neurotoxic tau aggregates [10]. However, persistent pathological stimuli may result in uncontrolled activation and senescence-like alterations of microglia, characterized by impaired phagocytic capacity of pathological substracts and sustained inflammatory responses [11]. Premature senescent microglia were found in the hippocampus of 6-month-old P301S mice, preceding AD symptoms and the onset of neurofibrillary tangle deposition [3]. Accordingly, targeting senescent microglia for elimination is considered a promising therapeutic strategy to counteract tau-mediated neuroinflammation and cognitive decline.

Irisin was initially discovered as an exercise-induced myokine in 2012 that was released into the blood following the proteolytic cleavage of fibronectin type III domain-containing protein 5 (FNDC5) in skeletal muscle, and it exerts a promoting effect on energy metabolism by driving white-to-brown adipose tissue conversion and boosting energy expenditure [12]. Intriguingly, irisin is also expressed in the human and mouse brains, and cerebrospinal fluid (CSF) [13–15].  Remarkably, peripheral irisin can cross the blood–brain barrier, leading to its elevation in CSF, thereby mediating exercise-induced cognitive benefits [13, 16]. Nonetheless, irisin levels declined in the hippocampus of AD patients and AD mouse models [13, 17, 18]. A recent study reported that peripheral irisin injection in transgenic tau mice can reduce tau phosphorylation and mitigate neuroinflammation, although its potential to reverse tau-induced cognitive deficits remains uncertain [19]. In addition, recent evidence indicates that irisin can suppress senescence phenotypes in vascular smooth muscle cells [20], cardiomyocytes [21], and nucleus pulposus cells [22] in aged mice. Based on the preliminary insights, we hypothesize that irisin could ameliorate neuroinflammation and cognitive impairment by averting microglial senescence and SASP in tauopathies.

In this study, we examined the alterations in irisin expression in the hippocampus of P301S mice and microglia BV2 cells incubated with tau K18 (a tau fragment containing all four repeats domain) for the first time. We further explored the therapeutic value of irisin for senescent microglia as well as the underlying mechanisms.

## Materials and methods

### Animals and drug administration

The P301S transgenic mice harboring human mutant microtubule-associated protein tau with a P301S mutation were initially sourced from the Model Animal Research Center of Nanjing University [23]. Female P301S mice and their wild-type (WT) littermates were raised in standard laboratory conditions under an artificial 12-hour light/dark cycle and had ad libitum access to food and water. The mice were randomly assigned into four groups: WT + vehicle group, WT + Irisin group, P301S + Vehicle group, and P301S + Irisin group. At the age of 6 months, mice in the irisin group received weekly intraperitoneal (IP) injections of 250 µg/kg irisin for three months, while the vehicle control group received an equivalent volume of phosphate-buffered saline (PBS). Animal experiments were approved by the institutional animal care ethical review board at Tongji Medical College, Huazhong University of Science and Technology.

### Preparation of K18 fibrils

Tau K18 is a fragment of full-length human tau containing four microtubule-binding repeats domain. The recombinant His-tagged K18 tau proteins were purified using a well-established protocol [24]. Briefly, Escherichia coli BL21(DE3) E. coli -expressed recombinant K18 protein was purified through a cation exchange chromatography column and subsequently eluted with sodium chloride dissolved in 125 mM imidazole. Then, the purified K18 protein was induced to generate pre-formed fibrils (PFFs) as described [25, 26]. Before being added to the culture medium, the K18 fibrils were sonicated for 30 min in a water bath sonicator.

### Cell culture and treatment

BV2 microglia were procured from Procell Life Science & Technology Co., Ltd. (Wuhan, China). The cells were cultured in a specific medium for BV2 cells (Catalog no.: CM-0493 A, Procell, China) and maintained in a humidified incubator at 37 °C with 5% CO2. To mimic the chronic tau stimulation in vitro, BV2 cells were exposed to 140 ng/mL K18 fibrils treatment for 72 h [24, 27]. In some experiments, BV2 cells were incubated with recombinant irisin (Catalog no.: HY-P70664, MedChemExpress, USA) at the indicated concentrations for 1 h before K18 fibrils were added to explore the potential therapeutic effects of irisin on microglial senescence.

### Western blotting

The protein samples were loaded onto 10–12% SDS-PAGE gel and electrophoresed for separation. The gel was then transferred to the nitrocellulose membrane, which was blocked with 5% bovine serum albumin (BSA) for 1 h at room temperature. Subsequently, the membrane was incubated overnight at 4℃ with the primary antibodies, as listed in Supplementary Table 1. After washing with TBS-Tween20 (TBST), the membrane was incubated with the horseradish peroxidase (HRP) -conjugated secondary antibody for 1 h at room temperature. Detection was performed using the ECL Imaging System (Clinx Science Instruments Co., Ltd.).

### Oxygen consumption rate

The measurement of cellular oxygen consumption rates (OCR) was conducted using Seahorse XF96 Extracellular Flux Analyzer (Agilent Technologies, USA) as described [28, 29]. One day before the assay, drug-treated or siRNA-treated BV2 cells were seeded on Seahorse Cell Culture plates at a density of 10,000 cells/well. At the same time, the cartridge plate was hydrated using 200 µL of XF calibrant buffer in a non-CO2 incubator at 37 °C overnight. OCR was measured under basal conditions and after the sequential injection of 1.5 µM oligomycin A, 1.5 µM fluoro-carbonyl cyanide phenylhydrazone (FCCP), and 0.5 µM antimycin A and rotenone cocktail. After the assays, cells were harvested by trypsinization and counted using Luna™ automated cell counter (Logos Biosystems, South Korea). OCR values were normalized to cell number.

### SA-β-gal staining

Senescence-associated β-galactosidase (SA-β-gal) staining was carried out using an SA-β-gal staining kit (Catalog no.: C0602, Beyotime, China) according to the manufacturer’s protocol [30]. Ten random imaging fields were collected per well, and SA-β-gal-positive cells were counted under the Leica DMi8 microscope (Germany).

### Golgi staining

Golgi staining was carried out using the commercial FD Rapid Golgi Stain Kit (FD Neurotechnologies, Beijing, China) [31]. Following the manufacturer’s protocol, the mouse brains were sequentially immersed in solutions A, B, and C. Afterward, the brain samples were sectioned into 100 μm slices using a vibratome (Leica, Germany). The brain sections were further immersed in combined Solutions D and E for 10 min. After a series of alcohol dehydration and xylene clearing, the sections were assembled under a coverslip. Spinal morphology was then visualized and captured using a Nikon optical microscope (Nikon, Japan).

### Statistical analysis

Statistical analysis was performed using GraphPad Prism software. The data were obtained from a minimum of three independent experiments. All data were tested and met the normal distribution and variance homogeneity criteria. For comparisons between two groups, an unpaired, two-tailed Student’s t-test was utilized, while comparisons among multiple groups were analyzed via one-way ANOVA or two-way ANOVA followed by Tukey’s post hoc test. A *p*-value of < 0.05 was considered statistically significant.

## Results

### Decreased irisin levels and increased microglial senescence in tauopathies

**Fig. 1:**
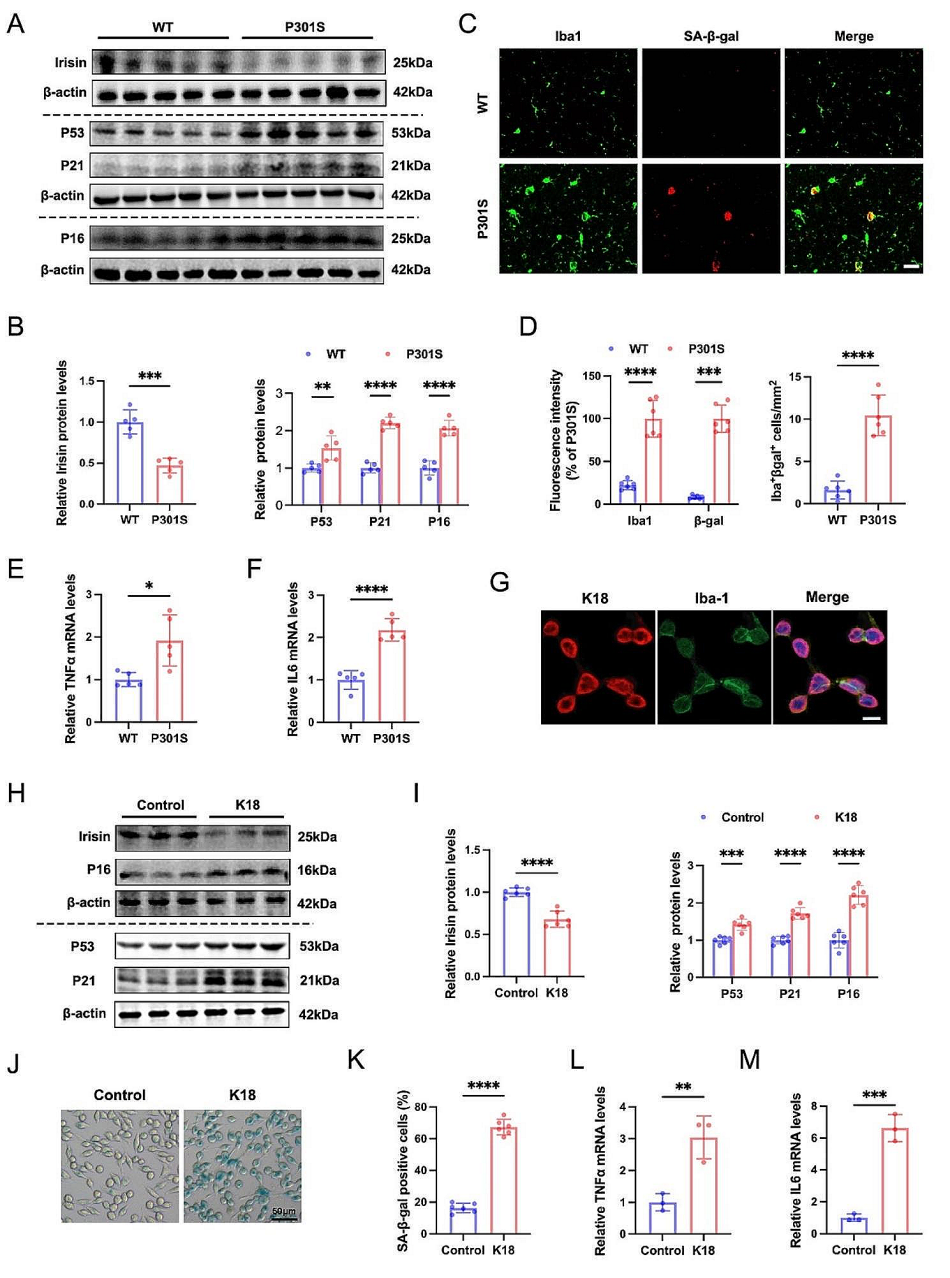
Decreased irisin levels and increased microglial senescence in tauopathies. (**A**-**B**) Representative western blots (**A**) and quantifications (**B**) of irisin, P53, P21, and P16 protein levels in the hippocampus from 9-month-old P301S mice and aged-matched WT mice. *N* = 5 mice for each group. (**C**-**D**) Representative images (**C**) and statistical quantification (**D**) of immunofluorescent analysis of Iba1 (green) and β-gal (red) in the hippocampus from 9-month-old P301S mice and aged control WT mice. Paraffin brain sections were immunostained with antibodies against Iba1 and β-gal and counterstained with DAPI to show nuclei (blue). Fluorescence intensity and positive number of Iba1^+^ βgal^+^ cells per mm^2^ were quantified. Scale bar, 20 μm. *N* = 6 mice for each group. (**E**-**F**) qRT-PCR analysis of TΝFα and IL6 mRNA expression in hippocampus from 9-month-old WT mice and P301S mice. *N* = 5 mice for each group. (**G**) Representative immunofluorescence images showing endocytosis of tau K18 protein in BV2 cells. BV2 cells were treated with His-tagged K18 protein for 6 h and then stained with anti-His antibody (Red) and anti-Iba1 antibody (Green). Scale bar, 10 μm. (**H**-**I**) Representative western blots (**H**) and quantitative analysis (**I**) for irisin, P53, P21, and P16 protein levels in control-treated or K18-treated BV2 cells. BV2 cells were subjected to either PBS or K18 fibril treatment for a duration of 72 h to replicate the in vitro milieu of chronic tau stimulation. *N* = 6 independent experiments for each group. (**J**-**K**) Representative images of SA-β-gal staining (**J**) and quantification of SA-β-gal positive BV2 cells (**K**). *N* = 6 independent experiments. (**L**-**M**) qRT-PCR analysis of TΝFα and IL6 mRNA expression in control-treated or K18-treated BV2 cells. *N* = 3 independent experiments. Data were expressed as mean ± standard deviation (SD). *p < 0.05, **p < 0.01, ***p < 0.001, ****p < 0.0001

An analysis of publicly accessible RNA sequencing datasets from human brain samples has demonstrated a decline in hippocampal FNDC5 mRNA, which encodes the precursor protein of irisin, with aging and tau pathology in the brain [18]. In this study, we first examined the changes in irisin levels in tau P301S transgenic mice. Western blot analysis revealed a significant reduction in irisin levels in the hippocampus of 6-month-old and 9-month-old P301S mice compared to age-matched WT mice (Fig. 1A-B, SFig. 1A-B). Subsequently, to further delve into the impact of tauopathies on microglia, we examined the expression levels of irisin in tau K18-induced microglial BV2 cells and vehicle-treated control cells. Tau K18, a fragment of full-length human tau, was utilized as it serves as a well-established model for tau due to its analogous physiological and pathological properties, such as microtubule binding and its capacity to form paired helical filaments (PHFs) [32–34]. To visualize K18 fibril uptake in BV2 cells, we subjected the cells to a 6-hour incubation with His-tagged K18 protein, followed by staining with anti-His antibody (Red) and anti-Iba1 antibody (Green) (Fig. 1G). Consistent with in vivo experiments, our results demonstrated a decreasing trend in irisin levels in BV2 cells exposed to K18 fibrils (Fig. 1H-I).

Building upon recent research emphasizing the critical involvement of microglial senescence in the progression of tauopathies [5, 35, 36], our study aimed to investigate the senescent-like characteristics of microglia in tauopathies. Western blot analysis unveiled a significant upregulation of P53, P21 and P16 expression in the hippocampus of P301S mice and K18-treated BV2 cells (Fig. 1A-B and H-I). Immunofluorescence staining further illustrated a notable rise in the densities of β-gal and Iba1, along with the percentage of SA-β-positive microglia (Iba1^+^) in the hippocampus of P301S mice (Fig. 1C-D). Correspondingly, a higher percentage of BV2 cells exhibited SA-β-gal activity in the presence of K18 fibrils (Fig. 1J-K). Moreover, our investigations revealed the induction of SASP in the hippocampus of P301S mice and BV2 cells following chronic exposure to K18 fibrils, and the secretion of SASP factors TNF-α and IL-6 was elevated both in vivo and in vitro (Fig. 1E-F and L-M). In conclusion, these results indicate a reduction in irisin levels and an escalation in senescence-like alterations of microglia in tauopathies, underscoring the pivotal role of irisin in tau-mediated microglial senescence and neuroinflammation.

### Irisin attenuates microglial senescence in vivo and in vitro

**Fig. 2:**
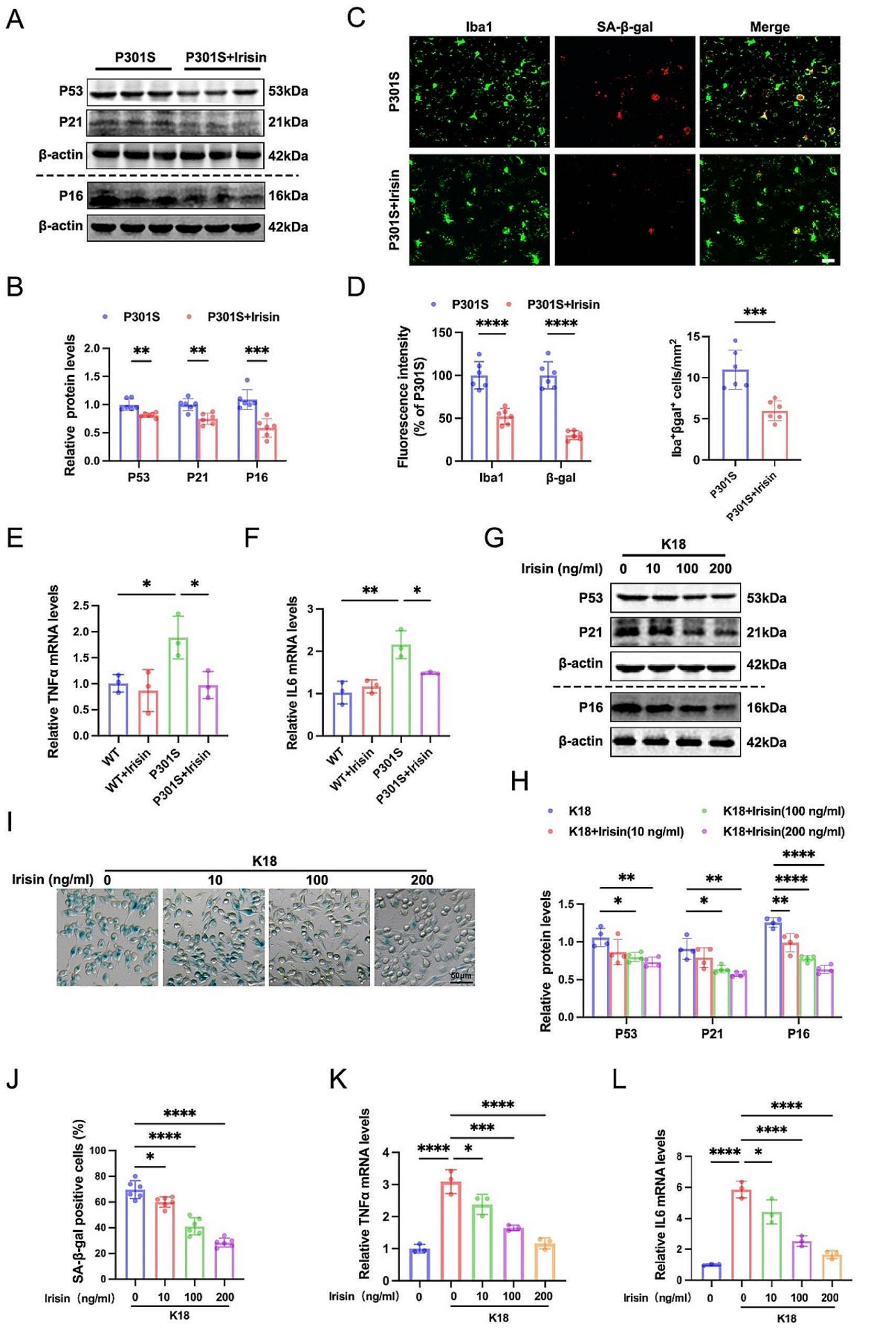
Irisin attenuates microglial senescence and senescence-associated secretory phenotype (SASP) in vivo and in vitro. (**A**-**B**) Representative western blots (**A**) and quantitative analysis (**B**) of P53, P21, and P16 protein levels in the hippocampus from P301S mice treated with irisin or vehicle control. *N* = 6 mice for each group. (**C**-**D**) Representative images (**C**) and statistical quantification (**D**) of immunofluorescent analysis of Iba1 (green) and β-gal (red) in the hippocampus from P301S mice treated with irisin or vehicle control. Fluorescence intensity and positive number of Iba1^+^ βgal^+^ cells per mm^2^ were quantified. Scale bar, 20 μm. *N* = 6 mice for each group. (**E**-**F**) qRT-PCR analysis of TΝFα and IL6 mRNA expression in hippocampus from P301S mice treated with irisin or vehicle control. *N* = 3 mice for each group. (**G**-**H**) Representative western blots (**G**) and quantitative analysis (**H**) of P53, P21, and P16 protein levels in BV2 cells treated with 0, 10, 100, and 200 ng/ml irisin and 140 ng/ml K18 fibrils. *N* = 4 independent experiments. (**I**-**J**) Representative images of SA-β-gal staining (**I**) and quantification of SA-β-gal positive BV2 cells (**J**). Scale bar, 50 μm. *N* = 6 independent experiments. (**K**-**L**) qRT-PCR analysis of TΝFα and IL6 mRNA expression in BV2 cells. *N* = 3 independent experiments. Data were expressed as mean ± SD. *p < 0.05, **p < 0.01, ***p < 0.001, ****p < 0.0001

Intermittent administration of low-dose irisin injections has previously showcased its beneficial effects [37, 38]. Consequently, we performed weekly IP injections of 250 µg/kg irisin in 6-month-old P301S mice for three months to evaluate the impact of irisin treatment on tau-induced microglial senescence and SASP. Western blotting analysis showed that irisin treatment significantly reduced P53, P21, and P16 levels in the hippocampus of P301S mice (Fig. 2A-B). Immunofluorescence analysis provided additional validation for a significant reduction in the proportion of SA-β-gal-positive microglia (Iba1^+^) in the hippocampus of irisin-treated P301S mice (Fig. 2C-D). Moreover, irisin treatment prevented tau-induced neuroinflammation, as evidenced by decreased TNF α and IL6 mRNA levels (Fig. 2E-F). These in vivo data indicated that the administration of irisin can alleviate the senescence-like phenotype of microglia. To investigate the potential therapeutic effects of irisin on microglial senescence in vitro, we preincubated BV2 cells with the indicated concentration of irisin for 1 h, followed by treatment of K18 fibrils for three days. As anticipated, irisin treatment prevented the K18-induced increase in P53, P21, and P16 expression, SA-β-gal activity, and TNF α and IL6 mRNA levels in a dose-dependent manner (Fig. 2G-L).

Microglial immunological function is essential not only for the secretion of inflammatory mediators but also for the clearance of pathological protein aggregates [39]. To investigate the potential of irisin in enhancing microglial-mediated tau clearance, we evaluated levels of total tau and phosphorylated tau in the hippocampus in P301S mice. Western blot analysis demonstrated a significant reduction in the expression levels of total tau (Tau5) and phosphorylated tau (pS404 and pS396) following irisin treatment (SFig. 2 A-B). Immunofluorescent staining further showed that irisin treatment markedly reduced the immuno-positive staining of phosphorylated tau (pS404) (SFig. 2 C). Taken together, these data suggest that irisin preserves microglial functionality against senescence-like phenotype, thereby attenuating neuroinflammation and tau pathology.

### Irisin improves cognitive impairments and synaptic dysfunction in P301S mice

**Fig. 3:**
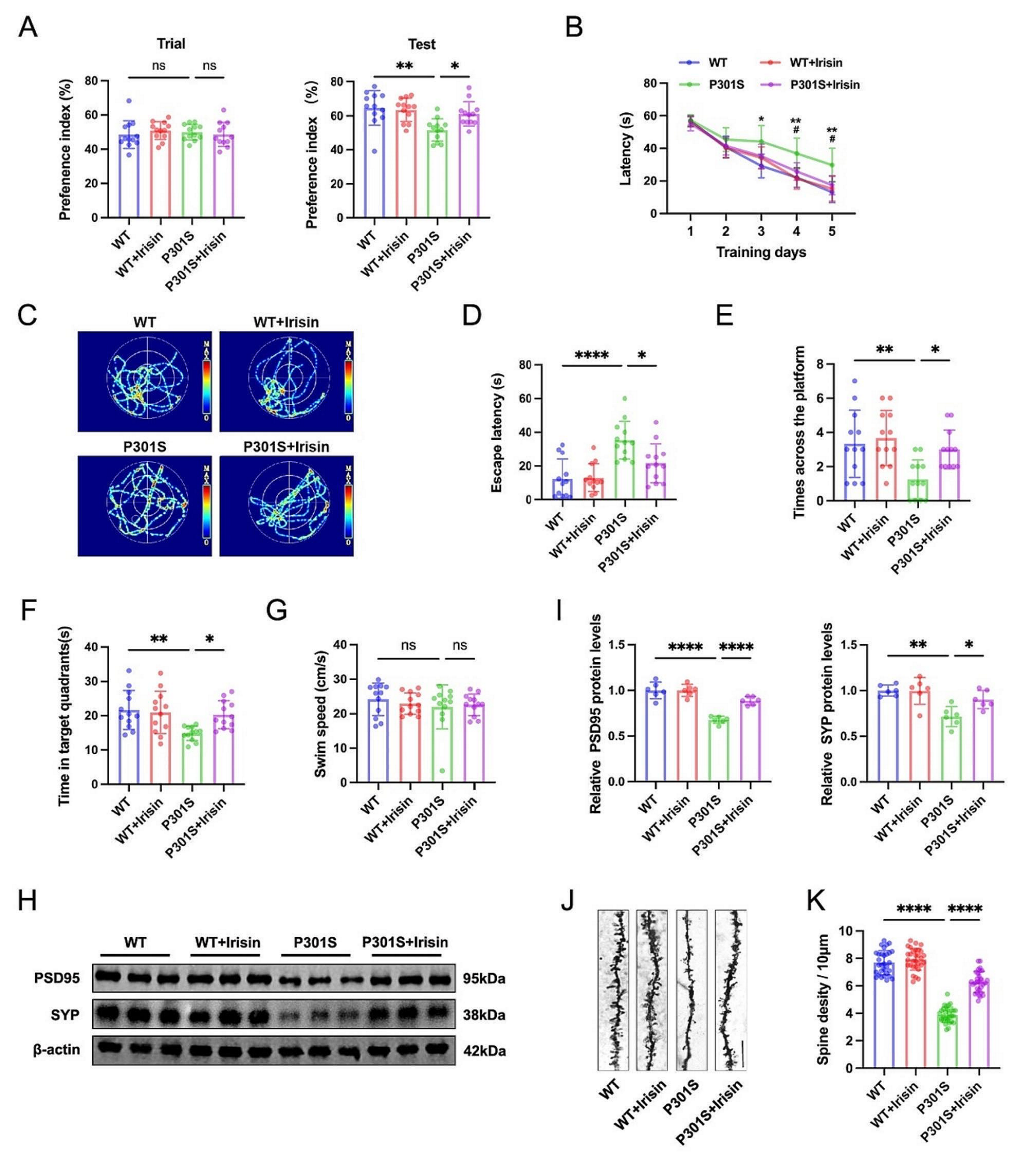
Irisin improves memory deficit and synaptic dysfunction in P301S mice. (**A**) Novel object recognition (NOR) test showed the preference index for WT and P301S mice with or without irisin treatment during training and testing phases. *N* = 12 mice for each group. (**B-G**) Morris water maze (MWM) test was performed to evaluate the spatial learning and memory abilities in WT and P301S mice with or without irisin treatment. Latency to find the hidden platform during 5 training trials (**B**). Representative swimming track (**C**), latency first entrance to target (**D**), target crossing times (**E**), duration in zone (**F**), and swimming speed (**G**) of mice on the probe test day. *N* = 12 mice for each group. (**H-I**) Representative western blots (**H**) and quantitative analysis (**I**) of postsynaptic density protein 95 (PSD95) and Synaptophysin (SYP) protein levels in the hippocampus. *N* = 6 mice for each group. (**J-K**) Representative images of Golgi staining (**J**) and quantitative analysis (**K**) of spine density in the hippocampal neurons. Scale bar, 10 μm. *N* = 30 neurons from 3 mice for each group. Data were presented as mean ± SD. **B**: *p < 0.05, **p < 0.01 (WT vs. P301S);^#^*p* < 0.05 (P301S vs. P301S + Irisin). Others: *p < 0.05, **p < 0.01, ***p < 0.001, ****p < 0.0001

A series of behavioral tests were conducted to assess the impact of irisin on cognitive function. In the test phase of the NOR test, P301S mice displayed memory deficits, as evidenced by a decreased recognition index for the novel object. At the same time, irisin treatment reversed the memory deficits in P301S mice, restoring their ability to recognize the novel object (Fig. 3A). During the 5-day MWM training phase, P301S mice exhibited learning deficits shown by longer latency to find the hidden platform than controls. In contrast, irisin administration reduced latency to find the platform in P301S mice (Fig. 3B). On the 7th day, spatial memory was assessed by removing the platform. We found that irisin administration dramatically rescued the spatial memory deficits of P301S mice, evidenced by a decreased latency to reach the previous platform site, increased target platform crossings, and increased time spent in the target quadrant (Fig. 3C-F). Swimming speed was comparable among all four groups, precluding the possibility of motor deficits (Fig. 3G).

Next, we measured synaptic density, synaptic protein expression, and neuron numbers across the various experimental groups. Western blotting analysis demonstrated that irisin treatment ameliorated the reduction of tau-induced synaptic-associated proteins (Fig. 3H-I). Golgi staining revealed a lower spine density in hippocampal neurons of P301S mice compared with the control group; however, irisin administration effectively rescued this reduction, restoring the spine density (Fig. 3J-K). An intact neuron is a neuron that displays visible nuclei, distinct nucleolus, and cytoplasmic Nissl bodies. Additionally, Nissl staining showed fewer intact neurons in the CA3 and DG region of P301S mice compared with the WT controls, which was effectively reversed by irisin administration (S Fig. 3A-B). Taken together, these findings offer compelling evidence that irisin treatment effectively reverses cognitive impairments and synapse loss in P301S mice.

### Irisin attenuates tau-induced mitochondrial dysfunction

**Fig. 4:**
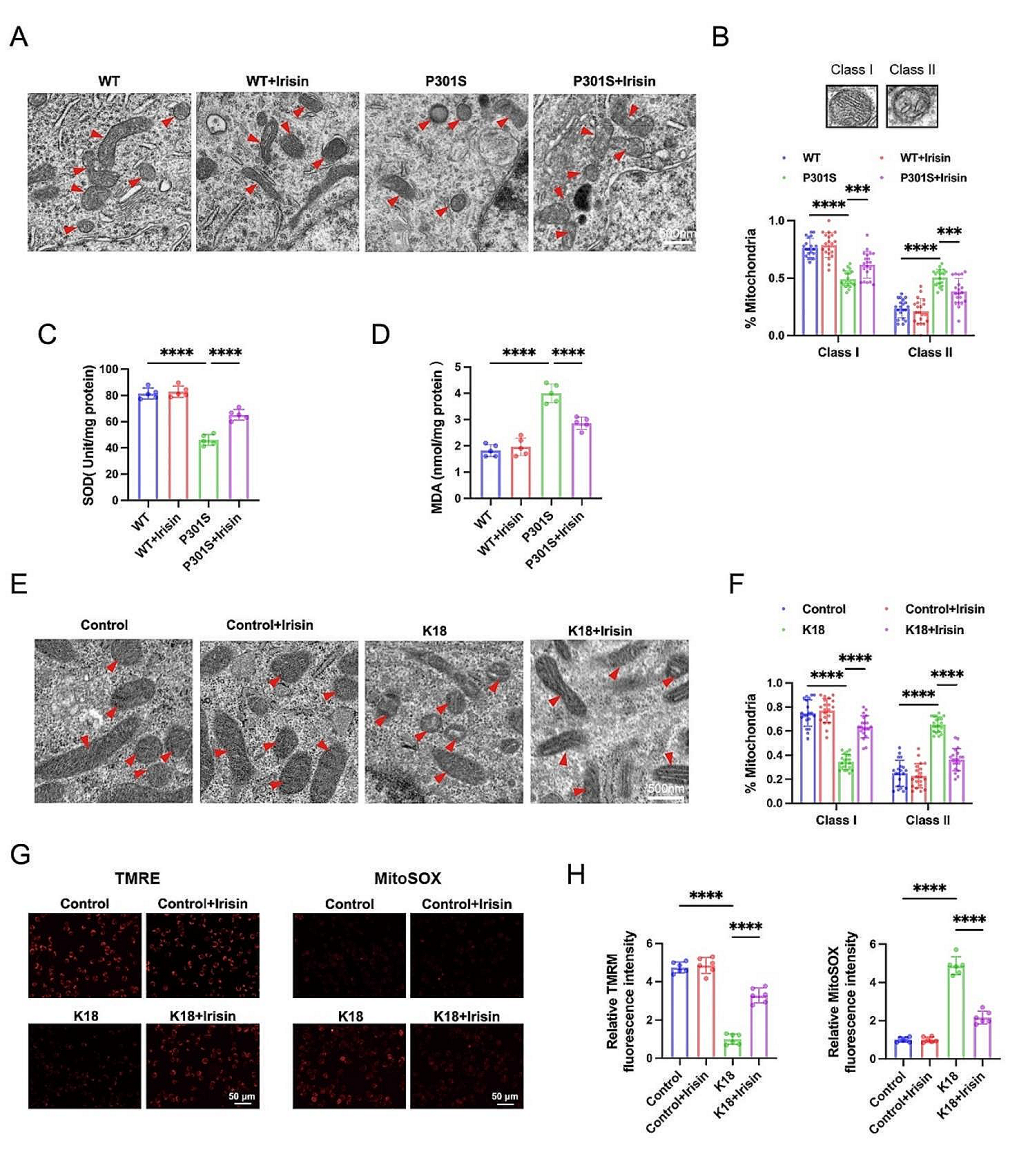
Irisin attenuates tau-induced mitochondrial dysfunction. (**A-B**) Representative transmission electron (TEM) micrographs showing mitochondrial morphology in the hippocampus (Α) and the proportion of each type of mitochondria (**B**). Class I: mitochondria with intact cristae; Class II: swollen mitochondria with vacuolation in the cristae. Scale bar, 500 nm. *N* = 20 analyzed fields from 3 mice for each group. (**C-D**) SOD activity and MDA contents in the hippocampus. *N* = 5 mice for each group. (**E-F**) Representative TEM micrographs showing mitochondrial morphology in BV2 cells (**E**) and the proportion of mitochondria subclasses (**F**). Scale bar, 500 nm. *N* = 20 analyzed fields from 3 independent experiments. (**G-H**) Representative images (**G**) and quantitation (**H**) of TMRE and MitoSox Red fluorescence in BV2 cells. *N* = 6 independent experiments. Data were expressed as mean ± SD. *p < 0.05, **p < 0.01, ***p < 0.001, ****p < 0.0001

To further investigate the underlying mechanisms by which irisin administration alleviates microglial senescence, we examined its effect on mitochondrial function. Transmission electron microscopy (TEM) was employed to analyze mitochondrial morphology, revealing mitochondrial swelling and the disappearance or vacuolization of mitochondrial cristae in the hippocampus of P301S mice (Fig. 4A-B). Similarly, K18-treated BV2 cells exhibited a drastic reduction of “active” class I mitochondria and a concomitant increase in “damaged” mitochondria class II compared with control cells (Fig. 4E-F). However, irisin treatment attenuated K18-induced mitochondrial damage in BV2 cells (Fig. 4E-F).

Moreover, we observed that irisin treatment significantly rescued the decreased SOD activity and the increased MDA levels in the hippocampus of P301S mice (Fig. 4C-D). We also measured mitochondrial membrane potential (MMP) by potentiometric probe TMRE and the mitochondrial reactive oxygen species (ROS) levels by MitoSOX fluorescent dye in BV2 cells. Our observations revealed that tau K18 resulted in a decrease in MMP and an increase in mitochondrial ROS production, both of which were attenuated by irisin treatment (Fig. 4G-H). Altogether, these findings indicate that irisin mitigates mitochondrial dysfunction in P301S mice and K18-treated BV2 cells.

### Irisin induces TFAM expression and promotes mitochondrial energy metabolism

**Fig. 5:**
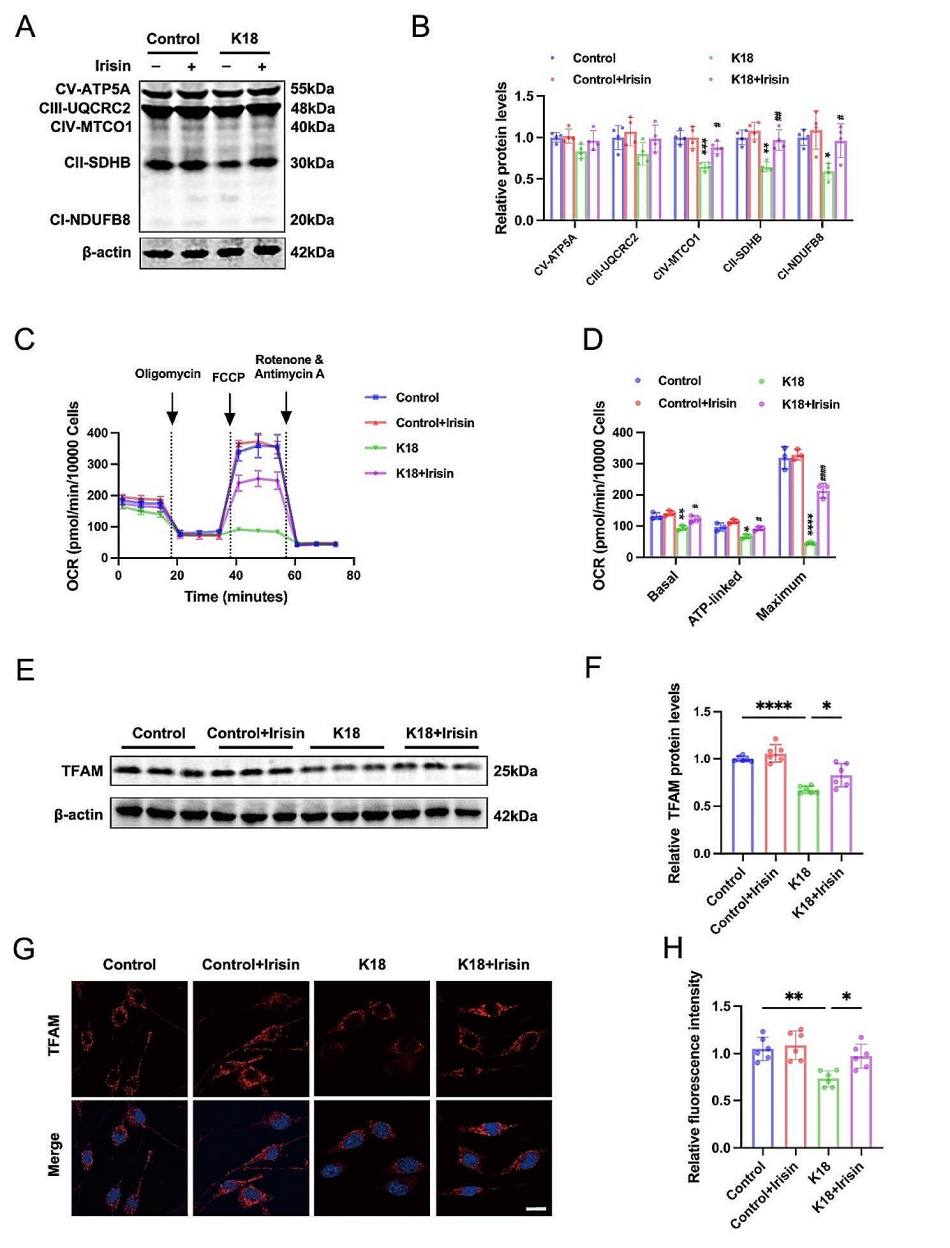
Irisin promotes TFAM expression and promotes OXPHOS in microglia. (**A-B**) Representative immunoblotting images (**A**) and quantitative analysis (**B**) of mitochondrial respiratory electron transport chain (ETC) subunits in BV2 cells. BV2 cells were preincubated with 200 ng/ml irisin for 1 h, followed by K18 fibrils (140 ng/ml) challenge for 72 h. *N* = 4 independent experiments. (**C-D**) Real-time changes in the oxygen-consumption rates (OCR) of BV2 cells (**C**). Basal respiration, ATP-linked respiration, and maximum respiration were calculated (**D**). *N* = 3 independent experiments. (**E-F**) Representative western blots (**E**) and quantitative analysis (**F**) of TFAM protein expression in BV2 cells. *N* = 6 independent experiments. (**G-H**) Representative immunofluorescence images (**G**) and quantitation (**H**) of TFAM in BV2 cells. Scale bar, 10 μm. *N* = 6 independent experiments. Data were presented as mean ± SD. **B**, **D**: *p < 0.05, **p < 0.01, ***, *p* < 0.001, vs. Control;^#^*p* < 0.05, ^##^*p* < 0.01, ^####^*p* < 0.0001, vs. K18; F, H: *p < 0.05, ***p < 0.001, ****p < 0.0001

Mitochondrial oxidative phosphorylation (OXPHOS) is the prominent mitochondrial function that meets the energy demand and combats oxidative damage. Furthermore, a decline in mitochondrial metabolism is a characteristic feature of aging cells. We assessed the protein expression of mitochondrial respiratory electron transport chain (ETC) subunits in BV2 cells to elucidate how irisin improves mitochondrial dysfunction. As decipted in Fig. 5A-B, the expression levels of the mitochondrial ETC subunits I, II, and IV showed a trend toward decrease, while the ETC subunits III and V levels remained unchanged. The administration of irisin upregulated the expression of ETC subunits I, II, and IV in K18-treated BV2 cells (Fig. 5A-B). Subsequently, we evaluated the microglial OXPHOS activity by measuring real-time O2 consumption rates (OCR) using the Seahorse XF96 Extracellular Flux Analyze. Comprehensive metabolic profiling unveiled that K18-treated BV2 cells exhibited reduced basal respiration, ATP-linked respiration, and maximum respiratory capacity, while irisin rescued K18-induced decline in the capacity of OXPHOS (Fig. 5C-D).

Mitochondrial transcription factor A (TFAM) plays a crucial role in regulating the biogenesis of the mitochondrial respiratory chain by triggering transcription and replication of mitochondrial DNA (mtDNA), which encodes 13 core ETC subunits [39, 40]. To explore how irisin improved mitochondrial energy metabolism, we examined TFAM protein levels. Western blot analysis revealed a reduction in TFAM levels in K18-induced BV2 cells (Fig. 5E-F). Notably, irisin administration significantly upregulated the TFAM protein levels in K18-treated BV2 cells (Fig. 5E-F). Immunofluorescence further confirmed that irisin augmented TFAM expression in K18-treated BV2 cells (Fig. 5G-H). These findings indicate impaired mitochondrial energy metabolism in tau-induced microglial senescence and suggest that irisin promotes mitochondrial OXPHOS by upregulating TFAM expression.

### Irisin enhances mitochondrial biogenesis and suppresses cellular senescence in a TFAM-dependent manner

**Fig. 6:**
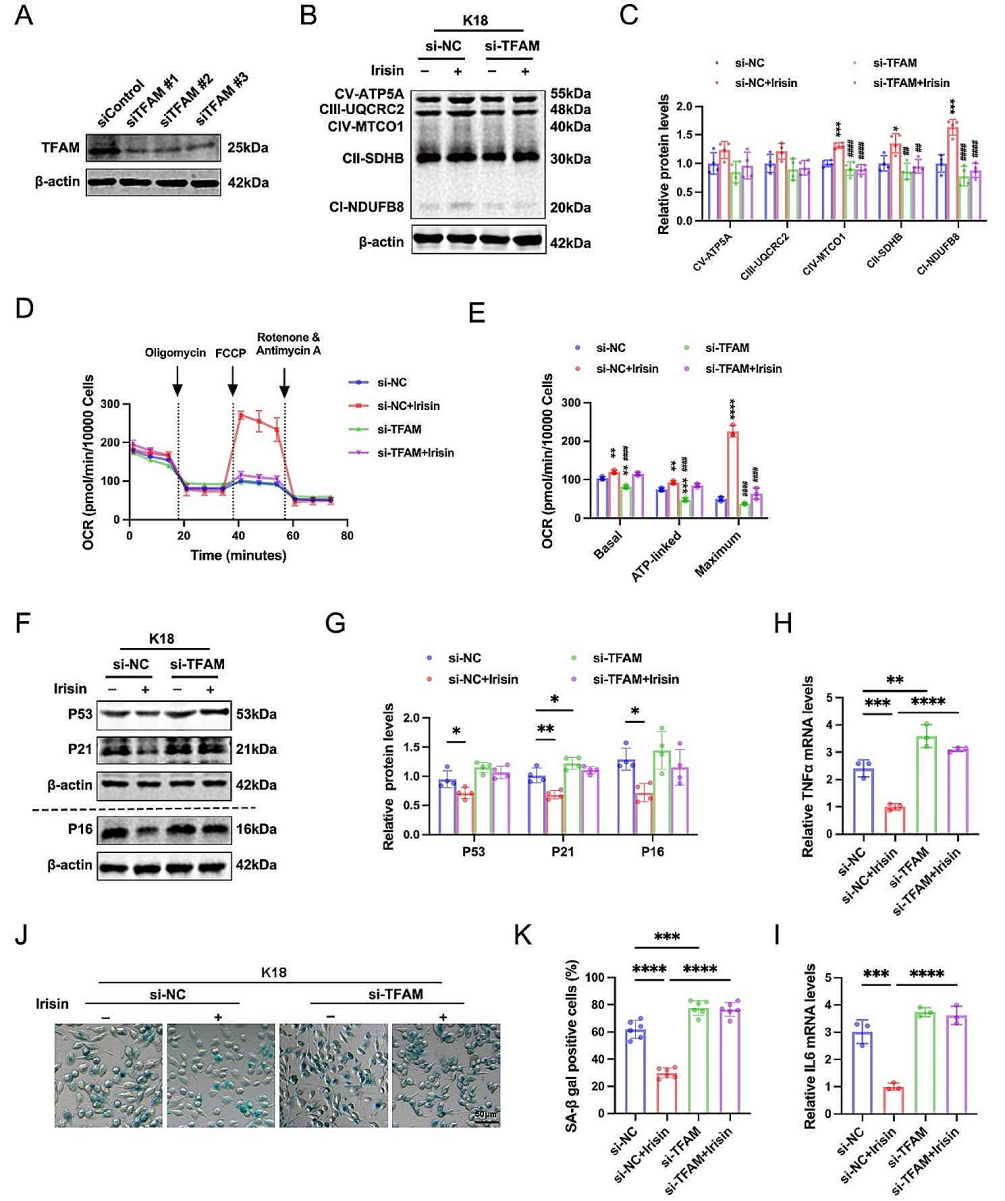
Irisin promotes OXPHOS and inhibits cellular senescence in a TFAM-dependent manner. (**A**) Western blot analysis showing knockdown efficiencies of three independent TFAM siRNAs. (**B-C**) Representative immunoblotting images (**B**) and quantitative analysis (**C**) of mitochondrial respiratory electron transport chain (ETC) subunits in BV2 cells. BV2 cells were transfected with control siRNA or TFAM siRNA for 24 h and then pre-incubated with vehicle or irisin (200 ng/ml) for 1 h followed by K18 fibrils stimulation for 72 h. *N* = 4 independent experiments. (**D-E**) Real-time changes in the oxygen-consumption rates (OCR) of BV2 cells in the indicated group (**D**). Basal respiration, ATP-linked respiration, and maximum respiration were calculated (**E**). *N* = 3 independent experiments. (**F-G**) Representative immunoblotting images (**F**) and quantitative analysis (**G**) of P53, P21, and P16 protein levels in BV2 cells. *N* = 4 independent experiments. (**H-I**) qRT-PCR analysis of TΝFα and IL6 mRNA expression in BV2 cells. *N* = 3 independent experiments. (**J-K**) Representative images of SA-β-gal staining (**J**) and quantification of SA-β-gal positive BV2 cells (**K**). *N* = 6 independent experiments. Data were presented as mean ± SD. **C**, **E**: **p < 0.01, *** *p* < 0.001, ****p < 0.0001, vs. si-NC;^##^*p* < 0.01, ^###^*p* < 0.001, ^####^*p* < 0.0001, vs. si-NC + Irisin. Others: * *p* < 0.05, *** *p* < 0.001, ****p < 0.0001

To investigate whether the effects of irisin on mitochondrial biogenesis and cellular senescence are mediated by TFAM, we conducted rescue experiments using TFAM siRNAs in vitro. The efficiency of TFAM knockdown by three independent siRNAs was confirmed via western blot analysis (Fig. 6A). To perform rescue experiments, BV2 cells were transfected with control siRNA or TFAM siRNA #1 for 24 h. Subsequently, the cells were pre-incubated with either vehicle or irisin (200 ng/ml) for 1 h, followed by stimulation with K18 fibrils for 72 h. As depicted in Fig. 6B-C, the irisin-induced upregulation of ETC subunit I, II, and IV protein expression levels was reversed when TFAM was silenced in BV2 cells. Moreover, the OCR results showed that TFAM knockdown abolished the restorative effect of irisin on mitochondrial OXPHOS impairment (Fig. 6D-E). In addition, the inhibitory effect of irisin on microglial senescence was abrogated by TFAM knockdown, evidenced by increased P53, P21, and P16 levels, SA-β-gal activity, and SASP components (Fig. 6F-K). These findings collectively indicate that irisin enhances mitochondrial bioenergetics and reduces senescence-like alterations of microglia in a TFAM-dependent manner.

### Irisin regulated TFAM activity via SIRT1/PGC1α signaling

**Fig. 7:**
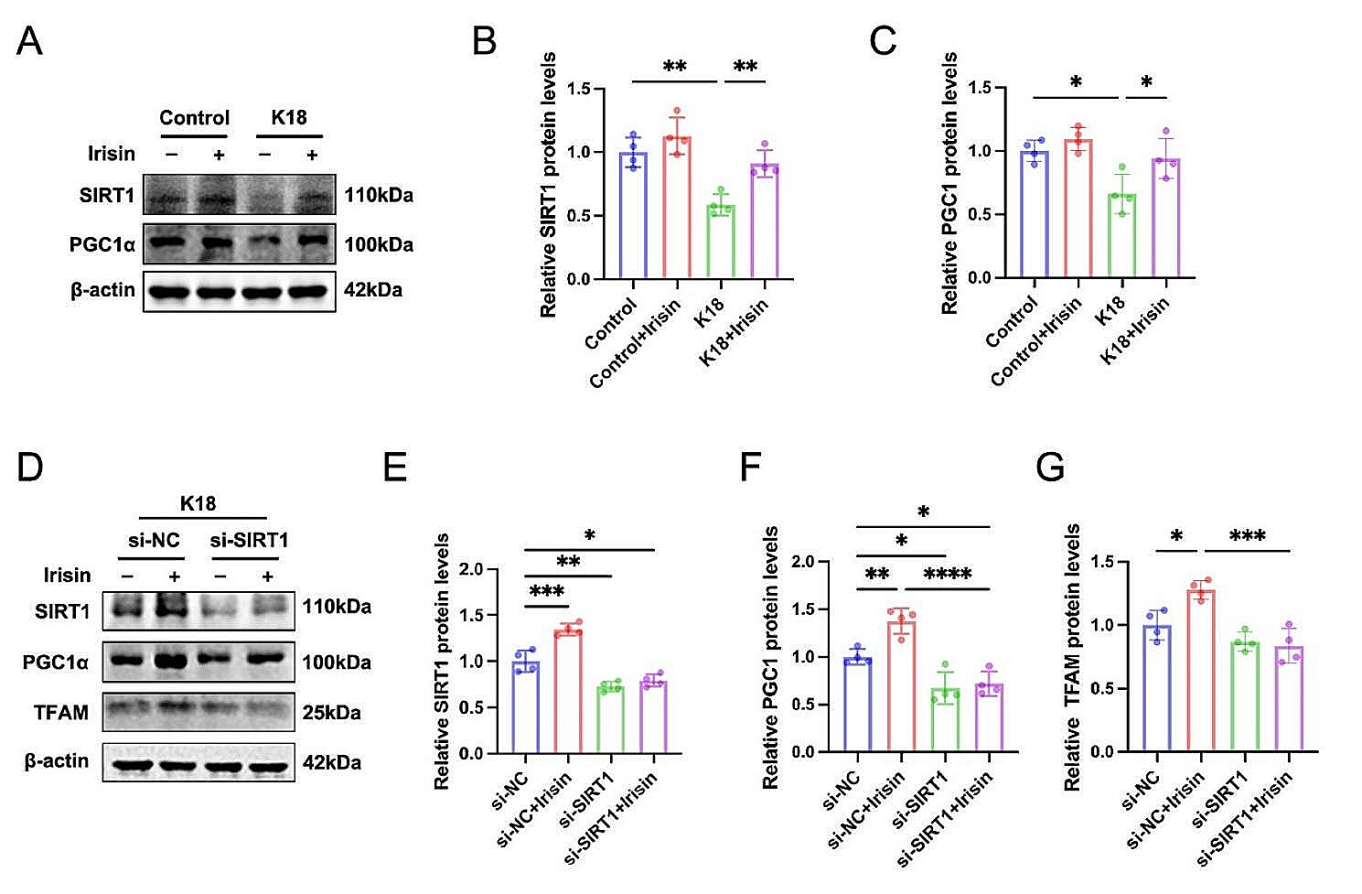
Irisin regulates TFAM activity via SIRT1/PGC1α signaling in microglia. (**A-C**) Representative western blots (**A**) and quantifications (**B, C**) of SIRT1 and PGC1α in the vehicle or K18-treated BV2 cells with or without irisin incubation. *N* = 4 independent experiments. (**D-G**) Representative western blots (**D**) and quantifications (**E-G**) of SIRT1, PGC1α, and TFAM in BV2 cells. BV2 cells were transfected with control siRNA or SIRT1 siRNA for 24 h, and then cells were preincubated with vehicle or irisin (200 ng/ml) followed by K18 fibrils stimulation for 72 h. *N* = 4 independent experiments. Data were expressed as mean ± SD. *p < 0.05, **p < 0.01, ***p < 0.001, ****p < 0.0001

After establishing irisin’s role in improving mitochondrial energy metabolism and eliminating senescence-like alterations of microglia by activating TFAM, we next sought to explore the mechanisms underlying irisin-mediated TFAM upregulation. Sirtuin1 (SIRT1) is an NAD^+^-dependent nuclear deacetylase known for its involvement in the deacetylation of histones and non-histone proteins. One of its crucial functions is the regulation of the activation of proliferation-activated receptor-gamma coactivator alpha (PGC1α) by deacetylating multiple lysine residues on it [41–43]. PGC-1α is a well-known transcriptional coactivator that regulates the transcription of nuclear respiratory factors (NRFs), thus promoting the expression of the TFAM [44]. Therefore, we aimed to investigate the potential role of the SIRT1/PGC1α signaling pathway in irisin-mediated TFAM upregulation. Our results demonstrated that SIRTI and PGC1α expression was reduced by K18 treatment compared with vehicle-controls, while both SIRT1 and PGC1α expression were upregulated in the K18 + Irisin group compared to the K18 group (Fig. 7A-C). However, SIRT1 knockdown hindered the activation of PGC1α and TFAM protein expression by irisin (Fig. 7D-G). Collectively, these results indicate that irisin activates TFAM expression by SIRT1/PGC1α signaling pathways in microglia.

## Discussion

**Fig. 8:**
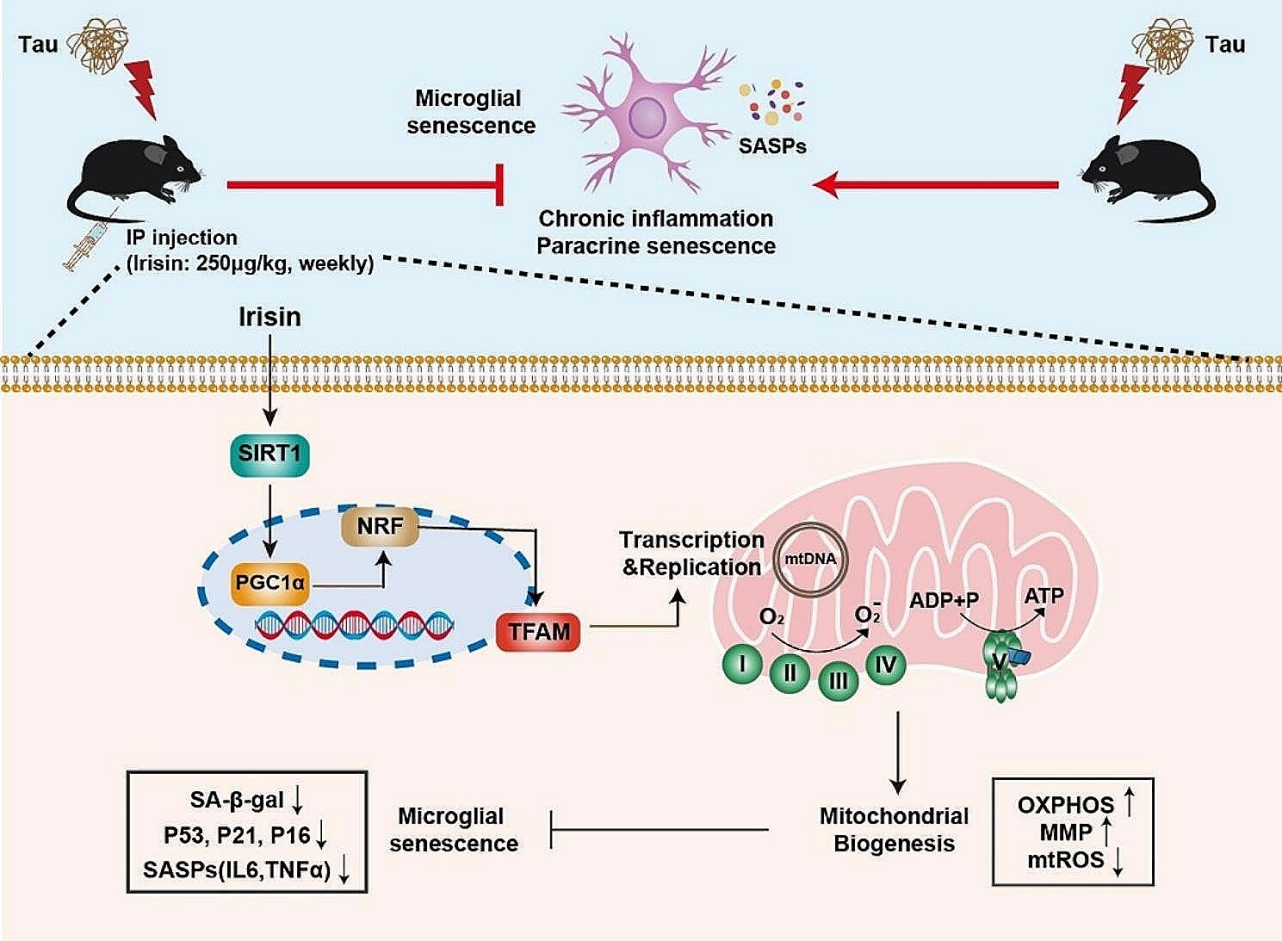
Schematic diagram of the effect of irisin on tau-induced microglial senescence. In tauopathies, irisin effectively prevents microglial senescence by promoting mitochondrial oxidative phosphorylation (OXPHOS) via TFAM, which encodes 13 crucial subunits of the mitochondrial respiratory electron transport chain (ETC) essential for OXPHOS. The upregulation of TFAM by irisin is thought to be mediated through the induction of the SIRT1/PGC1α signaling pathway

The study of glial senescence in AD has garnered significant interest [4, 25, 35, 45], especially following the detection of senescent glia accumulations in the brain of P301S transgenic mice in 2018 [3]. A recent investigation focusing on different tau mouse models highlighted the utility of P301S mice as an effective model for exploring brain cellular senescence, contrasting with the less suitable nature of P301L mice or 3xTg-AD mice [5]. Microglia exposed to chronic tau burden are more prone to adopt a senescent-like phenotype, leading to sustained secretion of the SASP and the formation of other senescent cells through paracrine signaling. In preclinical models of aging, chemotherapeutics, including senolytics that selectively target senescent cells for clearance and senomorphics that inhibit the pro-inflammatory senescent secretome, have demonstrated potential in extending lifespan and enhancing physical function [46–48]. In this study, we report, for the first time, that intermittent low-dose irisin injections effectively reversed tau-induced microglial senescence and cognitive decline in P301S mice. Mechanistically, we elucidated that irisin activated TFAM expression via the SIRT1/PGC1α signaling pathway, consequently enhancing mitochondrial metabolism and suppressing microglial senescence (Fig. 8).

Mitochondrial dysfunction has emerged as a crucial factor among the myriad complex factors contributing to cellular senescence [49]. Notably, it’s widely recognized in the medical community that pathological tau significantly impacts mitochondrial function through various mechanisms, e.g., directly interacting with mitochondrial proteins and thereby compromising their function and integrity, or disrupting mitochondrial quality control mechanisms, further contributing to mitochondrial dysfunction [50, 51]. Mitochondria is an active energy-producing organelle that relies heavily on ETC and OXPHOS. As immune cells, microglia undergo metabolic reprogramming in response to acute insults, shifting from OXPHOS to glycolysis to meet the high energy demands of processes like phagocytosis, cytokine secretion, and migration. However, previous studies have shown that chronic β-amyloid exposure leads to a broad metabolic defect in microglia, affecting both glycolysis and OXPHOS [28, 52]. Unlike acute stimulation, AD is a chronic disease. Therefore, we speculate that the microglia also exhibit multiple energy metabolism deficiencies in a chronic environment of pathological tau protein aggregation, ultimately leading to mitochondrial dysfunction and cellular senescence. Consistent with previous studies, we observed significant mitochondrial impairment in the hippocampus of P301S mice and K18-treated BV2 cells. Furthermore, we examined the OCR value of senescent BV2 cells and observed a significant reduction in mitochondrial OXPHOS. However, treatment with irisin reversed this impairment. Further investigation of glycolytic capacity or β-oxidation of fatty acids is necessary to characterize tau-tolerant microglia’s metabolic profile and explore the metabolic difference between β-amyloid-tolerant and tau-tolerant microglia.

The biogenesis of the respiratory chain is a complex process that requires the coordinated expression of the nuclear and mitochondrial genome [53]. TFAM, a member of the high mobility group box protein family, plays a crucial role in this process by binding upstream of two major promoters of the mitochondrial genome (known as the light- and the heavy-strand promoter) to promote the transcription and replication of mtDNA, which in turn encodes 13 crucial ETC subunits [39, 40]. Reduced expression of TFAM has been observed in the hippocampus of APPswe/PS1dE9 transgenic mice and AD patient brains [54–56]. Genetic upregulation of TFAM has been shown to improve the activities of complex I and IV, reduce lipid peroxidation accumulation, and rescue age-related learning and memory deficits in aged mice [57]. However, to the best of our knowledge, there is limited research on TFAM protein levels and the therapeutic role of TFAM overexpression in tauopathies. In this study, we demonstrated that tau K18 inhibited the expression of TFAM in BV2 cells while irisin upregulated the expression of TFAM in K18-treated BV2 cells. Irisin promoted mitochondrial biogenesis and inhibited cellular senescence in a TFAM-dependent manner, as silencing TFAM eliminated the inhibitory effect of irisin on microglial senescence and the restorative effect of irisin on mitochondrial OXPHOS.

SIRT1, a member of the mammalian sirtuin family, regulates diverse biological processes such as gene transcription, metabolism, and cell senescence [42]. Multiple lines of evidence supported the neuroprotective role of SIRT1 in neurodegeneration [58–60]. Activation of SIRT1 may trigger biological responses by deacetylating PGC1α, leading to the stabilization and increased transcriptional activity of PGC1α [42]. PGC1α, a well-known transcription coactivator, governs the transcriptional expression of genes related to mitochondrial biogenesis [44]. Notably, previous study suggests that PGC1α can positively regulate TFAM expression [44]. Herein, a performance of the SIRT1/PGC1α pathway was noted following K18 fibrils and irisin treatment in BV2 cells. We found a significant reduction in SIRT1 and PGC1α expression in BV2 cells exposed to K18, which was reversed by irisin. SIRT1 inhibition by siRNA resulted in the downregulation of PGC1α and TFAM protein levels, suggesting that TFAM induction may be mediated by SIRT1/PGC1α signaling.

Despite the discovery of the inhibitory effect of irisin on microglia senescence, this study still has several limitations. Firstly, our research was geared towards deciphering the anti-senescence mechanisms by which irisin operates in microglia. However, given irisin’s pleiotropic effects and its potential to impact a range of central nervous system (CNS) cells, such as astrocytes and infiltrating macrophages, we concede that our in vivo observations may encompass more than just the mitigation of microglial senescence. Future work endeavors are necessitated to illuminate the breadth and specificity of irisin’s modulatory influence on senescence across various cellular constituents in tauopathies. Secondly, this study utilized BV2 cells, a mouse microglial cell line, to investigate microglial function, but results from experiments using primary microglia are considered more reliable. Thirdly, while the regimen involving weekly intraperitoneal injections of 250 µg/kg irisin demonstrated satisfactory therapeutic effects in P301S mice, it would be more compelling to test different dosage gradients and dosing intervals to determine the optimal treatment regimen.

In conclusion, we demonstrated the presence of senescence-like alterations of microglia in the hippocampus of P301S mice and K18-treated microglial BV2 cells. The expression of exercise hormone irisin was decreased in tauopathies. Intermittent low-dose injections of exercise hormone irisin improve cognitive impairments and synaptic dysfunction in P301S mice. Mechanistically, irisin mitigated the microglial senescence by enhancing mitochondrial OXPHOS in a TFAM-dependent manner. Our findings suggest that irisin may represent a promising therapeutic strategy for inhibiting microglial senescence and neuroinflammation in tauopathies.

### Electronic supplementary material

Below is the link to the electronic supplementary material.

Supplementary Material 1

## Data Availability

The datasets utilized in this study can be obtained from the corresponding author upon reasonable request.

## Abbreviations

AD: Alzheimer’s disease
ETC: Electron transport chain
FNDC5: Fibronectin type III domain-containing protein 5
OCR: Oxygen consumption rates
OXPHOS: Oxidative phosphorylation
PGC1α: Proliferation-activated receptor-gamma coactivator alpha
PSD95: Postsynaptic Density protein 95
SA-β-gal: Senescence-associated β-galactosidase
SASP: Senescence-associated secretory phenotype
SYP: Synaptophysin
TFAM: Mitochondrial transcription factor A
