# Supplementary material for: Irisin inhibits microglial senescence via TFAM-mediated mitochondrial metabolism in a mouse model of tauopathy
Source: Immun Ageing. 2024 May 14;21:30. doi: 10.1186/s12979-024-00437-0 (PMC11092051; doi:10.1186/s12979-024-00437-0)
Supplement: Supplementary file 1 — Supplementary Material 1 [file 12979_2024_437_MOESM1_ESM.pdf]

Supplementary Information

Irisin inhibits microglial senescence via TFAM-mediated mitochondrial metabolism in a mouse model of tauopathy

Cailin Wang[1,#], Xiufeng Wang[1,#], Shangqi Sun[1], Yanmin Chang[1], Piaopiao Lian[1], Hongxiu Guo[1], Siyi Zheng[1], Rong Ma[2,*], Gang Li[1,*]

Supplementary Figures

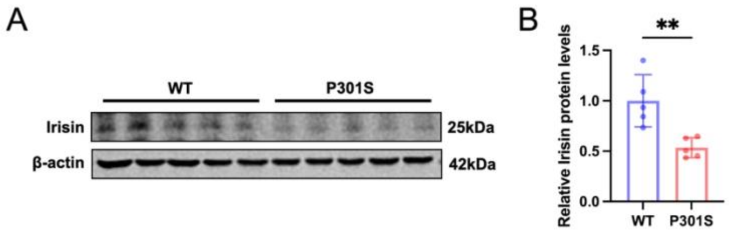

Supplementary Figure 1. Decreased irisin levels in hippocampus from 6-month-old P301S mice. (A-B) Western blots (A) and quantifications (B) of irisin protein levels in the hippocampus from 6-month-old P301S mice and aged-matched WT mice. N = 5 mice for each group.

Data were presented as mean ± SD. **p < 0.01

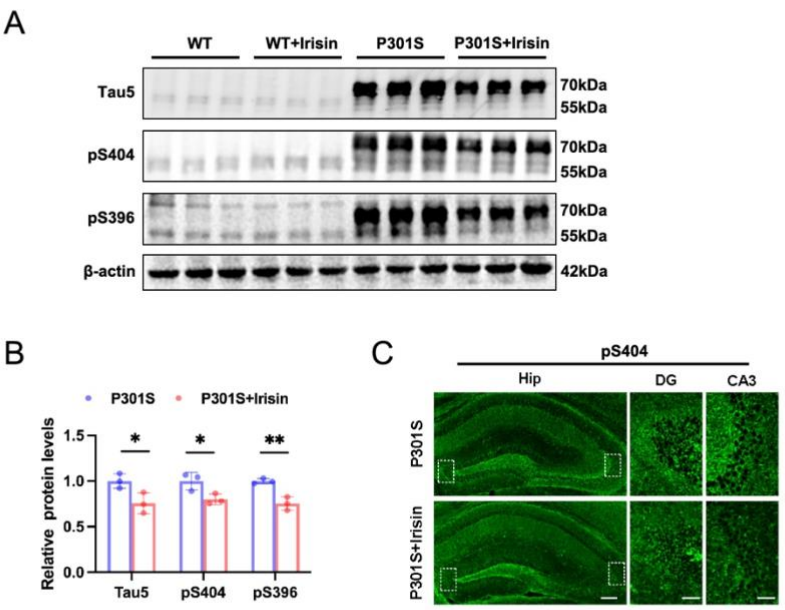

Supplementary Figure 2. Irisin reduces tau pathology in P301S mice.

(A-B) Western blots (A) and quantifications (B) of total tau (Tau5) and phosphorylated tau (pS404, and pS396) in the hippocampus. N = 3 mice for each group.

(C) Representative fluorescence images of phosphorylated tau (pS404) in the hippocampus of P301S (Hip: hippocampus; DG: dentate gyrus.). Scale bars: 200 μm (whole hippocampus), 50 μm (CA3 and DG).

Data were presented as mean ± SD. *p < 0.05, **p < 0.01

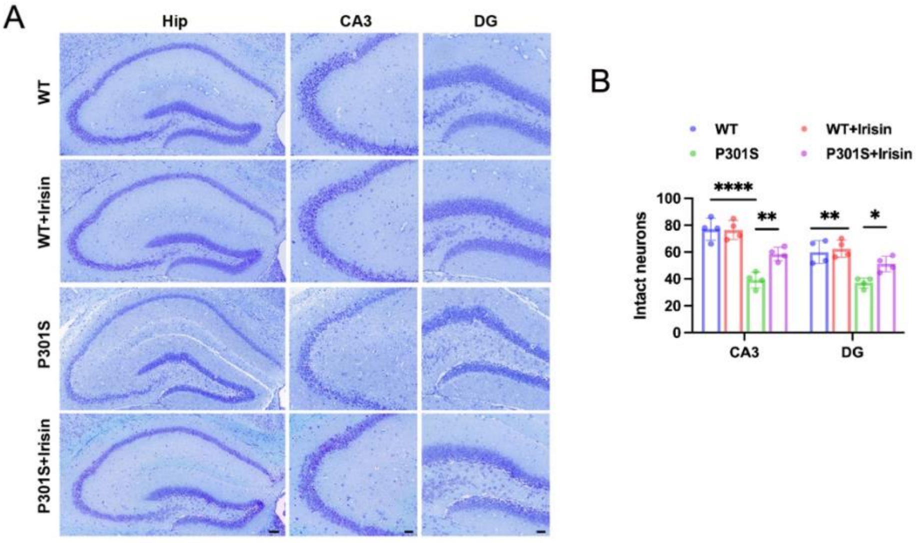

Supplementary Figure 3. Irisin restores neuronal loss in the hippocampus from P301S mice.

(A-B) Representative Nissl staining pictures of the hippocampus (A) and quantitation of Nissl-stained neurons of CA3 and DG (dentate gyrus) areas (B). Left-hand panels show the whole hippocampus, scale bar, 100 μm; middle- and right-hand panels show CA3 and DG areas, scale bar, 50 μm. N =4 mice for each group.

Data were presented as mean ± SD. *p < 0.05, **p < 0.01, ****p < 0.0001

Materials and methods

1. Small interfering RNA transfection

All small interfering RNAs (siRNAs) and negative controls (NCs) were synthesized by Gene Create (Wuhan, China). For transfection, cells were seeded in six-well plates to reach 60%–70% confluence. The siRNAs were transfected into BV2 cells using Lipofectamine 6000 (Beyotime, China).

Mouse siRNA sequences are as follows:

Scrambled siRNA sense (5′- UUCUCCGAACGUGUCACGUTT -3′), antisense (5′- ACGUGACACGUUCGGAGAATT -3′).

si-TFAM -1 sense (5- GCUGAGUGGAAAGCAUACAAATT -3), antisense (5′- UUUGUAUGCUUUCCACUCAGCTT -3′);

si-TFAM -2 sense (5′- GGGUAGCUAUCCAAAGAAACCTT -3′), antisense (5′- GGUUUCUUUGGAUAGCUACCCTT -3′);

si-TFAM -3 sense (5′- AGGAUUCGUUACGACAAUGAATT -3′), antisense (5′- UUCAUUGUCGUAACGAAUCCUTT -3′);

si-SIRT1 sense (5′- GCACUAAUUCCAAGUUCUATT -3′), antisense (5′- UAGAACUUGGAAUUAGUGCTT -3′).

2. Quantitative real-time PCR (qRT-PCR)

According to the manufacturer's protocol, total RNA from animal tissues and cells was extracted using the Total RNA Extraction Kit (Catalog no.:SM132-02, Seven, China). Reverse transcription and subsequent qRT-PCR experiments were performed as described (30). The cycle quantification

(Cq) values of each target gene were determined using StepOne 2.3 software. GAPDH served as an internal standard. The following primers were used: TNFα forward 5′-TGACTCCAAAGTAGACCTG-3′, reverse 5′- ACACTCACAAACCACCAA-3′; IL-6 forward 5′-ATTTCCTCTGGTCTTCTGG -3′, reverse 5′- TGGCTTTGTCTTTCTTGTTA-3′; GAPDH forward 5′- GGTGAAGGTCGGTGTGACCG -3′, reverse 5′- CTCGCTCCTGGAAGATGGTG -3′.

3. Immunofluorescence staining

Immunofluorescence staining was performed on mouse brain sections and cultured cells. The mice were transcardially perfused with PBS, and brains were isolated and fixed in 4% paraformaldehyde (PFA). The brain tissues were then dehydrated, embedded in paraffin, and sliced into 4 μm thick sections. The paraffin-embedded brain sections were dewaxed and hydrated for immunofluorescence, followed by heat-mediated antigen retrieval. BV2 cells were seeded on coverslips and fixed with 4% PFA for 10 min. Afterward, coverslips and sections were blocked in 5% BSA at room temperature for 1 h and subsequently incubated with primary antibodies overnight at 4°C. Then, samples were incubated with Alexa Fluor 488- or Cy3-conjugated goat anti-rabbit/mouse secondary antibodies (Servicebio, China) for 1 h at room temperature, followed by counterstaining with DAPI. Images were visualized by fluorescence microscopy (Olympus, VS120, Japan). The following primary antibodies for immunostaining were used: rabbit anti-IBA1 (Catalog no.: 019-19741, Wako, Japan), mouse anti-IBA1 (Catalog no.: GB12105, Servicebio, China), mouse anti-β Galactosidase (Catalog no.: 66586-1-Ig, Proteintech, China), mouse anti-His (Catalog no.: 66005-1-Ig, Proteintech, China), rabbit anti-TFAM (Catalog no.: ab252432, Abcam, USA), rabbit anti- Tau(Phospho-Ser404) (Catalog no.: 11112, Signalway, USA).

4. Transmission electron microscopy

For transmission electron microscopy (TEM), overnight, BV2 cell and hippocampal tissue samples were collected and fixed with 2.5% glutaraldehyde at 4 °C. The fixed samples undergo infiltration treatment with 1% osmium tetroxide solution, followed by dehydration using a series of ethanol concentrations and embedding in epoxy resin. Subsequently, ultra-thin sections of 50 nm thickness are prepared using a vibrating knife. These sections are then mounted on copper grids and stained

with uranyl acetate and lead citrate dyes. The mitochondrial morphology was observed under the Hitachi H-7000 electron microscope.

5. Detection of MMP and mitochondrial ROS

Mitochondrial membrane potential (MMP) and mitochondrial reactive oxygen species (ROS) were measured by tetramethyl rhodamine methyl ester staining (TMRM; Catalog no.: C2001S, Beyotime, China) and mitoSOX staining (Catalog no.: M36008, Thermo Fisher Scientific, United States), respectively. In brief, cells were plated on the confocal dish and incubated with 1X TMRM for 30 min or 2.5 μM MitoSOX for 10 min at 37 °C. After washing to remove unbound dyes, the fluorescence was observed under a fluorescence microscope (Leica, DMi8, Germany).

6. Measurement of SOD activity and MDA content

The superoxide dismutase (SOD) activities and malondialdehyde (MDA) levels of hippocampus tissues were measured using SOD Assay Kit (Catalog no.: S0103, Beyotime, China) and Lipid Peroxidation MDA Assay Kit (Catalog no.: S0131S, Beyotime, China) according to the manufacturer's instructions, respectively. The absorbance values for SOD and MDA were determined at wavelengths of 450 nm and 532 nm, respectively, utilizing a Synergy2 multi-mode microplate reader (BioTek, USA). The SOD activity and the MDA content were normalized to the total protein.

7. Nissl staining

Paraffin-embedded brain sections were first deparaffinized in xylene and rehydrated in a descending alcohol series. These prepared sections were subsequently stained using Nissl staining solution (Catalog no.: G1036, Servicebio, China) for 5 min. After staining, the sections were dried and covered with neutral resin. Stained slides were scanned with Pannoramic SCAN slide scanner 250 (3D Histech, Hungary).

8. Behavioral tests

Behavioral experiments, including novel object recognition (NOR) and the Morris water maze (MWM), were conducted to evaluate the cognitive capacity as described (32).

The NOR test is a learning and memory assessment method based on animals' innate tendency to explore new things. The mice were first acclimatized to an object-free box (50 × 50 × 50cm) for a 5-min habituation 24 h before the test. During the training phase, the mice were introduced to two identical objects, A and A′, positioned at opposite ends of the box where they were allowed a 5-minute exploration period. Object A′ was substituted on the second day with a novel object B. The mice were put into the box again for another 5-minute exploration session. A video camera above the box recorded their exploratory behavior. The exploration time for objects A and B was denoted as TA and TB, respectively. The ratio TB / (TA + TB) was calculated to determine the preference for the novel object.

The MWM test assesses spatial learning and memory in experimental animals. During the spatial learning phase, mice were trained for consecutive 5 days to find a hidden platform below the water surface in a circular pool. The latency to reach the platform was recorded, and the animals were allowed to stay on the platform for 20 seconds. If the mice fail to find the platform within 60 seconds, they would be guided there and allowed to remain for 20 seconds. The spatial memory was assessed a day following the spatial learning phase. The platform was removed, and the mice were allowed to navigate the maze for 60 seconds. The motion trails of mice were recorded and analyzed using the MWZ-100 system (Techman, China)

Supplementary Table 1. Antibodies for WB used in this study

**Antibodies**	**Source**	**Catalog No.**
Anti-P53	CST, USA	2524
Anti-P21	Proteintech, China	28248-1-AP
Anti-P16	Abcam, USA	ab211542
Anti-P16	Santa, USA	Sc-1661
Anti-Irisin/FNDC5	Abcam, USA	ab174833
Anti-Tau5	Abcam, USA	ab80579

Anti-Tau (Phospho-Ser404)	Signalway Antibody, USA	11112
Anti-Tau (Phospho-Ser396)	Signalway Antibody, USA	11102
Anti-β-actin	Proteintech, China	20536-1-AP
Anti-PSD 95	Abclonal, China	A0131
Anti-Synaptophysin	Proteintech, China	17785-1-AP
Anti-TFAM	Abcam, USA	ab252432
Total OXPHOS Rodent WB Antibody Cocktail	Abcam, USA	ab110413
Anti-SIRT1	Proteintech, China	13161-1-AP
Anti-PGC1α	Abclonal, China	A12348
